# Hesitation about coronavirus vaccines in healthcare professionals and general population in Spain

**DOI:** 10.1371/journal.pone.0277899

**Published:** 2022-12-01

**Authors:** Francesc Saigí-Rubió, Hans Eguia, Albert Espelt, Salvador Macip, Marina Bosque-Prous

**Affiliations:** 1 Faculty of Health Sciences, Universitat Oberta de Catalunya (UOC), Barcelona, Spain; 2 SEMERGEN New Technologies Working Group, Madrid, Spain; 3 Department of Epidemiology and Public Health, Faculty of Health Sciences of Manresa, Universitat de Vic–Universitat Central de Catalunya (UVic-UCC), Manresa, Spain; 4 Centro de Investigación Biomédica en Red de Epidemiología y Salud Pública (CIBERESP), Madrid, Spain; 5 Departament de Psicobiologia i Metodologia en Ciències de la Salut, Universitat Autònoma de Barcelona (UAB), Bellaterra. Spain; 6 Department of Molecular and Cell Biology, Mechanisms of Cancer and Aging Laboratory, University of Leicester, Leicester, United Kingdom; Universitas Syiah Kuala, INDONESIA

## Abstract

**Background:**

This study attempts to provide a picture of the hesitancy to vaccination against COVID-19 in Spain during the 2021 spring-autumn vaccination campaign, both in the general population and in healthcare professionals.

**Methods:**

The participants were recruited using social media such as Facebook and Twitter, in addition to the cooperation of health personnel contacted with the collaboration of medical scientific societies. A cross-sectional study was carried out that included the response of an online questionnaire. The data were collected from April 30 to September 26, 2021. To assess the different associations between variables to be measured, we fit Poisson regression models with robust variance.

**Results:**

Responses were obtained from 3,850 adults from the general population group and 502 health professionals. Of the overall sample, 48.6% of participants from the general population were vaccinated against COVID-19, whereas in the healthcare professionals, 94.8% were vaccinated. The prevalence of general population vaccination increased with age, and was higher in women than men. Most participants did not show a preference for any vaccine itself. However, the prevalence of people vaccinated with their preferred vaccine was higher for the ones vaccinated with Pfizer’s vaccine. 6.5% of the general population reported being reticent to be vaccinated. People from younger age groups, people with lower educational levels and those who were not from a risk group showed greater reluctance to be vaccinated. No gender differences in reluctancy were found.

**Conclusions:**

Health professionals were significantly less likely to refuse vaccination even though they had more doubts about the safety and efficacy of vaccines. On the other hand, younger people, those with a lower level of education and those who were not from a risk group were the most hesitant.

## Introduction

Controlling the impact of the COVID-19 pandemic and reducing its health effects and the serious implications for economic growth and social development depend, to a great extent, on prevention efforts [1, 2]. In addition to the widespread use of masks, social isolation, and lockdowns, the development of a coronavirus vaccine has been a critical tool [3–7]. By March 2021, several COVID-19 vaccines had already been developed and vaccination efforts had started in many countries [8, 9], recognizing the fact that mass vaccination was a necessary step to curb the pandemic [10].

In Spain, the mass vaccination campaign began on 27th December 2020, as did the 27 EU countries. The first vaccine to be administered was the one developed by Pfizer/BioNTech (Comirnaty), followed by those developed by Moderna (Spikevax), AstraZeneca (ChAdOx1) and Janssen once they were approved by the European Medicines Agency [11]. According to the Spanish’s COVID-19 vaccination strategy, the eligibility criteria used to prioritize vaccination were, firstly, to vaccinate health professionals (mainly physicians, nurses, and those professionals working at nursing home residences) and the general population, divided by age groups, starting with those over 80 years old and those groups with greater vulnerability, and then gradually shifting to a younger age ranges [12].

Despite efforts to massively vaccinate against COVID-19, a significant proportion of the population was reluctant to be vaccinated. In fact, the issue of vaccine rejection has been so prevalent in the years prior to the COVID that even the WHO considered it as one of the top ten threats to global health since 2019, as evidenced by the resurgence of some infectious diseases [13–17]. One study conducted in Spain showed an acceptance rate of the COVID-19 vaccines of only of 33.7% in August 2020 and a progressive increase to 48.3% in December 2020 [18]. However, the acceptance rate in other studies with smaller samples showed that almost 74.33% of the general Spanish population surveyed in June 2020 supported vaccination [19], while between September 2020 and November 2020 the acceptance rate was 77.5% [20].

Several international studies carried out just before the start of the vaccination campaign have shown that the hesitation to get vaccinated against COVID-19 is due to various factors, such as a feeling of ineffectiveness, fear of adverse effects, a feeling of already being immune due to having survived the disease, insecurity due to a vaccine that has been commercialized in a record, fear of dying due to chronic diseases that that can be exacerbated by the vaccines, belief in the existence of nanorobots in the vaccine or simply being generically anti-vaccines [19–29]. In addition to the influence of these factors, in Spain it has also been described that the high percentage of indecision of the Spanish population could be partly explained by disinformation and lack of political consensus [18]. Being the press and radio, together with social media the most used sources of information by the Spanish population, the latter would be linked to false news or non-contrasted statements that could derive in an increase in the reluctance to get vaccinated [18, 30].

A similar situation was evident in the United Kingdom, where 7 out of 10 respondents were in favour of vaccination against the coronavirus [31]. However, a later study carried out in February 2021 in the United Kingdom while the national vaccination program was already ongoing showed that 86% of those unwilling or planning to reject the COVID-19 vaccine in December 2020 changed their minds and hoped to receive it as soon as possible [32]. To achieve this change, public health messages are thought to have played a very important role, as did the efforts of primary care physicians and primary care centres [18, 33–35].

As WHO recommends [36], it is important to periodically analyze the population’s attitudes towards vaccination, as it can be useful to define intervention measures to raise awareness and highlight the benefits and safety of vaccines, as well as to inform communication campaigns aimed to strengthen trust in health authorities [27, 37–39]. Healthcare professionals have a better understanding of the effects of vaccines than the general population, including the odds of complications and their mechanisms of action. Moreover, healthcare professionals are more exposed to pathogens and, therefore, are aware of their increased risk of being infected. Thus, we hypothesized that healthcare professionals and general population could differ in their vaccine hesitance due to these and other factors. For that reason, the aim of this study was to examine attitudes towards vaccines and intentions to vaccinate against COVID-19 according to sociodemographic characteristics for both general population and healthcare professionals after vaccine approval in Spain.

## Methods

The study uses a cross-sectional design with data from a brief questionnaire on COVID-19 vaccination created *ad hoc* for this study, which included questions about sociodemographic variables and information regarding COVID-19 vaccination status. The study population were adults living in Spain, which were divided into two groups depending on their occupation: general population and health professionals. A convenience sampling was used. For the general population, the questionnaire was delivered by social media (Twitter, Linkedin, Facebook and WhatsApp) and data were collected from April 30 to July 21 2021. The health professionals received the questionnaire by email through their professional associations, Twitter and Facebook. The questionnaire was available from April 30 to September 26 2021. The first screen of the online questionnaire contained the main information about the study and its objective and, before filling in the questionnaire, each participant had to give their consent to participate. In addition, to ensure the confidentiality of the results obtained, the questionnaires were anonymous, meaning that the participants could not be identified in any way what-soever.

The dependent variable was having been vaccinated against COVID-19 (yes; no). Other relevant variables in vaccinated people were the type of vaccine (Astra Zeneca; Pfizer; Moderna; Johnson & Johnson; others) and whether they were vaccinated with their preferred vaccine (yes; no; without any preferences). In people who were not vaccinated against COVID-19, other relevant variables were whether the person planned to be vaccinated (yes; no), the best vaccine in their opinion (Astra Zeneca; Pfizer; Moderna; Sputnik; Johnson & Johnson; without preference; others) and the main reason for not vaccinating against COVID-19 (COVID-19 is not a real disease; Not believing in the pandemic, it is a lie created by governments/pharmaceutical companies to make money; COVID is a new disease and the vaccine has not yet been proven to work/be effective; Concerned about the unknown serious effects of the vaccine in the future; Believing that it is not necessary to get vaccinated, better to get sick and generate antibodies; Other reasons). Besides, the sociodemographic variables analyzed in this study were group of age, gender, region of residence, educational level (only for the general population), profession (only for health professionals) and being a member of a COVID-19 risk group.

The study protocol was approved by the Universitat Oberta de Catalunya (UOC) Ethics Committee (Code: “20210308_fsaigi_Vacuna” and Date: April 21, 2021).

### Data analysis

First of all, we described the sociodemographic characteristics of the sample for both the general population and the health professionals. Then, we estimated the prevalence of vaccination against COVID-19 in the participants from the general population and the health professionals and for each sociodemographic variable. After that, in those people that reported being vaccinated against COVID-19, we estimated the overall proportion of people vaccinated with the type of vaccine of their preference (yes, no or without any preference) in the general population and in the health professionals group. We used Chi squared test to assess differences between each category of the variable “being vaccinated with their preferred vaccine” for each type of vaccine in both the general population and health professionals’ groups. Finally, we described for those who were not vaccinated the proportion who was planning to get vaccinated in the near future, their beliefs about the best vaccine (only in those planning to get vaccinated) and the main reasons for not wanting to get vaccinated (only in those who did not plan to get vaccinated against COVID-19 in the future). To ascertain the factors related with not wanting to get vaccinated, we estimated Poisson regression models with robust variance adjusting for the sociodemographic variables. This latter analysis was conducted only in the general population as only 9 health professionals reported this decision. We conducted all the analyses separately for the general population and the health professionals. All statistical analyses were conducted with STATA 16.

## Results

In our study, 3850 adults from the general population (GP) living in Spain and 502 healthcare professionals (HP) participated. Of the overall sample, 48.6% of participants from the GP were vaccinated against COVID-19, whereas in the HP, 94.8% were vaccinated.

Of the people from the GP interviewed, 66.9% were over 45 years of age. The HP interviewed were also mostly over 45 years old (65.4%). In general, most of the respondents were women (70.1% GP, 63.9% HP) and were not part of an at-risk group (67% GP, 48.6% HP). 75.6% of the GP had a university education while the majority of the HP interviewed were physicians (72.7%). Most of the GP interviewed were from the autonomous community of Catalonia, while the HP were mainly from the autonomous communities of Andalusia and the Canary Islands (Table 1).

**Table 1:** Characteristics of the study sample.

	General population			Health professionals		
	N	%	95%CI	N	%	95%CI
**Group of age**						
18–24 years	127	3.3	(2.8–3.9)	2	0.4	(0.1–1.6)
25–34 years	350	9.1	(8.2–10.0)	62	12.4	(9.7–15.5)
35–44 years	797	20.7	(19.5–22.0)	110	21.9	(18.5–25.8)
45–54 years	1072	27.8	(26.5–29.3)	147	29.3	(25.5–33.4)
55–64 years	907	23.6	(22.2–24.9)	150	29.9	(26.0–34.0)
≥65 years	597	15.5	(14.4–16.7)	31	6.2	(4.4–8.7)
**Gender**						
Man	1152	29.9	(28.5–31.4)	181	36.1	(32.0–40.4)
Woman	2698	70.1	(68.6–71.5)	321	63.9	(59.6–68.0)
**Region of residence**						
Andalusia	92	2.4	(2.0–2.9)	140	27.9	(24.1–32.0)
Aragon	21	0.5	(0.4–0.8)	13	2.6	(1.5–4.4)
Principality of Asturias	46	1.2	(0.9–1.6)	22	4.4	(2.9–6.6)
Balearic Islands	65	1.7	(1.3–2.1)	6	1.2	(0.5–2.6)
Canary Islands	7	0.2	(0.1–0.4)	154	30.7	(26.8–34.9)
Cantabria	12	0.3	(0.2–0.5)	3	0.6	(0.2–1.8)
Castile and Leon	58	1.5	(1.2–1.9)	11	2.2	(1.2–3.9)
Castilla—La Mancha	9	0.2	(0.1–0.4)	19	3.8	(2.4–5.9)
Catalonia	3250	84.4	(83.2–85.5)	36	7.2	(5.2–9.8)
Valencian Community	115	3.0	(2.5–3.6)	20	4.0	(2.6–6.1)
Extremadura	6	0.2	(0.1–0.3)	6	1.2	(0.5–2.6)
Galicia	50	1.3	(1.0–1.7)	14	2.8	(1.7–4.7)
Community of Madrid	82	2.1	(1.7–2.6)	25	5.0	(3.4–7.3)
Region of Murcia	8	0.2	(0.1–0.4)	22	4.4	(2.9–6.6)
Foral Community of Navarre	4	0.1	(0.04–0.3)	0		
Basque Country	20	0.5	(0.3–0.8)	9	1.8	(0.9–3.4)
La Rioja	4	0.1	(0.04–0.3)	2	0.4	(0.1–1.6)
Autonomous City of Melilla	1	0.03	(0.004–0.2)	0		
**Educational level**						
No education	10	0.3	(0.1–0.5)	NA		
Primary education	155	4.0	(3.4–4.7)	NA		
Secondary education	773	20.1	(18.8–21.4)	NA		
University education	2912	75.6	(74.3–77.0)	NA		
**Profession**						
Medical doctor	NA			365	72.7	(68.6–76.4)
Nurse	NA			104	20.7	(17.4–24.5)
Other health professionals	NA			33	6.6	(4.7–9.1)
**Member of a risk group**						
Yes	871	22.6	(21.3–24.0)	211	42.0	(37.8–46.4)
No	2581	67.0	(65.5–68.5)	244	48.6	(44.2–53.0)
No, but living with someone at-risk	398	10.3	(9.4–11.3)	47	9.4	(7.1–12.2)

The prevalence of vaccination in the GP was higher with increasing age, 83.6% (95%CI: 80.4–86.3) of GP over 65 years were vaccinated while only 18.9% (95%CI: 13–26.7) of those aged 18–24 years. In the HP there were no statistically significant differences between the different age groups. In the GP, men tended to be less vaccinated than women, while no statistically significant differences were observed according to the educational level in the GP. For the HP, there were no statistically significant differences in the prevalence of vaccination in both nurses and physicians. However, the prevalence of COVID-19 vaccination was lower in other HP [78.8% (95%CI: 61.7–89.6)] in comparison with the prevalence in physicians [96.4% (95%CI: 94–97.9)]. Finally, those who were part of an at-risk group showed a prevalence of 73.4% (95%CI: 70.3–76.2) of vaccination in the GP and 95.7% (95%CI: 92.0–97.8) in the HP group (Table 2).

**Table 2:** Prevalence of vaccination against COVID-19 depending on sociodemographic and health characteristics.

	General population				Health professionals			
	Vaccinated against COVID-19				Vaccinated against COVID-19			
	N	%	95%CI	p-value	N	%	95%CI	p-value
**Group of age**								
18–24 years	127	18.9	(13.0–26.7)	<0.001	2	100		0.562
25–34 years	350	32.3	(27.6–37.4)		62	96.8	(88.0–99.2)	
35–44 years	797	33.5	(30.3–36.9)		110	92.7	(86.1–96.3)	
45–54 years	1072	36.0	(33.2–38.9)		147	94.6	(89.5–97.3)	
55–64 years	907	64.2	(61.0–67.2)		150	96.7	(92.2–98.6)	
≥65 years	597	83.6	(80.4–86.3)		31	90.3	(73.9–96.9)	
**Gender**								
Man	1152	44.1	(41.3–47.0)	<0.001	181	94.5	(90.0–97.0)	0.793
Woman	2698	50.5	(48.6–52.4)		321	95.0	(92.0–96.9)	
**Educational level**								
No education	10	60.0	(29.7–84.2)	0.103	NA			
Primary education	155	50.3	(42.5–58.1)		NA			
Secondary education	773	44.8	(41.3–48.3)		NA			
University education	2912	49.5	(47.7–51.3)		NA			
**Profession**								
Medical doctor	NA				365	96.4	(94.0–97.9)	<0.001
Nurse	NA				104	94.2	(87.7–97.4)	
Other health professionals	NA				33	78.8	(61.7–89.6)	
**Member of a risk group**								
Yes	871	73.4	(70.3–76.2)	<0.001	211	95.7	(92.0–97.8)	0.722
No	2581	40.9	(39.0–42.8)		244	94.3	(90.5–96.6)	
No, líving with someone at-risk	398	44.2	(39.4–49.1)		47	93.6	(82.0–97.9)	
*Total*	*3850*	*48*.*6*	*(47*.*0–50*.*2)*		*502*	*94*.*8*	*(92*.*5–96*.*5)*	

In general, respondents did not have a preferred vaccine (65% in the GP and 54.1% in the HP group). Moreover, 21.2% of the GP and 44.2% of the HP were vaccinated with the vaccine they wanted, while 13.7% of the GP and 1.7% of the HP were vaccinated with a vaccine they did not prefer. As can be seen in Table 3, the type of vaccine with the highest acceptance rates in the GP was Pfizer’s vaccine. In Spain, almost all HP were vaccinated with BNT162b2’s vaccine, so the comparison of the acceptance rate among types of vaccine was not possible.

**Table 3:** Distribution of people vaccinated with their preferred vaccine according to the type of COVID-19 vaccine received.

		Vaccinated with their preferred vaccine						
		Yes		No		Without any preference		
	N	%	95%CI	%	95%CI	%	95%CI	P-value
**General population**	** **	** **	** **	** **	** **	** **	** **	
**Type of vaccine**								
Astra Zeneca	1058	4.4	(3.4–5.9)	22.6	(20.2–25.2)	73.0	(70.2–75.6)	<0.001
Pfizer	657	48.2	(44.4–52.1)	0.6	(0.2–1.6)	51.1	(47.3–55.0)	
Moderna	126	22.2	(15.8–30.3)	7.9	(4.3–14.1)	69.8	(61.3–77.2)	
Johnson and Johnson	9	11.1	(1.5–50.0)	11.1	(1.5–50.0)	77.8	(42.1–94.4)	
Others	9	22.2	(5.6–57.9)	11.1	(1.5–50.0)	66.7	(33.3–88.9)	
*Total*	*1859*	*21*.*2*	*(19*.*4–23*.*2)*	*13*.*7*	*(12*.*2–15*.*4)*	*65*.*0*	*(62*.*8–67*.*2)*	
**Health professionals**	** **	** **		** **		** **		
**Type of vaccine**								
Astra Zeneca	15	20.0	(6.6–47.1)	20.0	(6.6–47.1)	60	(34.7–80.9)	<0.001
Pfizer	428	46.5	(41.8–51.3)	0.9	(0.4–2.5)	52.6	(47.8–57.3)	
Moderna	29	20.7	(9.6–39.1)	3.4	(0.5–20.9)	75.9	(57.3–88.1)	
Johnson and Johnson		0.0		0.0		0.0		
Others	1	100.0		0.0		0.0		
*Total*	*473*	*44*.*2*	*(39*.*8–48*.*7)*	*1*.*7*	*(0*.*8–3*.*4)*	*54*.*1*	*(49*.*6–58*.*6)*	

As can be seen in Table 4, most of the respondents who were not vaccinated had plans to be vaccinated (86.6% of the GP and 61.5% of the HP). The vaccine they preferred was the Pfizer’s vaccine (34.4% of the GP and 62.5% of the HP). 46.8% of the GP and 31.3% of HP had no preference. In the group with reticence to be vaccinated against COVID-19, about 30% of the GP and the HP reported that their main reason for not getting vaccinated was that they were concerned about the unknown serious effects of the vaccine in the future. Other reasons reported less frequently can be seen in Table 4.

**Table 4:** Distribution of variables in people who had not been vaccinated.

	General population			Health professionals		
	n	%	95%CI	n	%	95%CI
**Plans to get vaccinated**						
Yes	1713	86.6	(85.0–88.0)	16	61.5	(41.1–78.6)
No	249	12.6	(11.2–14.1)	9	34.6	(18.5–55.3)
No data	17	0.9	(0.5–1.4)	1	3.8	(0.5–24.6)
**Best vaccine in their opinion** ^**a**^						
Astra Zeneca	16	0.9	(0.6–1.5)	0		
Pfizer	590	34.4	(32.2–36.7)	10	62.5	(35.7–83.4)
Moderna	122	7.1	(6.0–8.4)	0		
Sputnik	27	1.6	(1.1–2.3)	0		
Johnson & Johnson	40	2.3	(1.7–3.2)	1	6.3	(0.7–37.6)
Others	92	5.4	(4.4–6.5)	0		
Without preference	802	46.8	(44.5–49.2)	5	31.3	(12.6–58.9)
No data	24	1.4	(0.9–2.1)	0		
**Main reason for not vaccinating against COVID-19** ^**b**^						
COVID-19 is not a real disease. Not believing in the pandemic, it is a lie created by governments /pharmaceutical companies to make money.	17	6.8	(4.3–10.7)	1	11.1	(1.1–59.1)
COVID is a new disease and the vaccine has not yet been proven to work/be effective.	50	20.1	(15.5–25.5)	1	11.1	(1.1–59.1)
Concerned about the unknown serious effects of the vaccine in the future.	80	32.1	(26.6–38.2)	3	33.3	(8.9–71.9)
Believing that it is not necessary to get vaccinated, better to get sick and generate antibodies.	43	17.3	(13.0–22.5)	0		
Other reasons	45	18.1	(13.8–23.4)	4	44.4	(14.6–79.0)
No data	14	5.6	(3.3–9.3)	0		

^a^ Only asked to those who planned to get vaccinated in the future.

^b^ Only asked to those who do not plan to get vaccinated.

6.5% of the GP reported being reticent to be vaccinated. Table 5 shows the variables associated with vaccine hesitation. Lower prevalence of reticence in being vaccinated against COVID-19 was found in people aged 55–64 (aPR = 0.5; 95%CI: 0.27–0.94) and older than 65 years old (aPR = 0.42; 95%CI: 0.21–0.82) when comparing with the younger age group (18–24 years). A lower level of education was also a risk factor for reticence: when compared to university education, people with no education, primary education and secondary education had 4.11 (95%CI: 1.37–12.34), 1.75 (95%CI: 0.97–3.15) and 1.65 (1.24–2.19) higher prevalence of reticence to be vaccinated, respectively. Finally, not being part of a risk group was also associated with greater reticence to be vaccinated, having 1.69 (95%CI: 1.16–2.46) higher risk than those who were part of a risk group.

**Table 5:** Prevalence Ratios (PR) and adjusted Prevalence Ratios (aPR) of COVID-19 vaccine reticence in adults of the general population, estimated used Poisson regression models.

	N	% Vaccine reticence	PR	95%CI	P-value	aPR	95%CI	P-value
*Total*	*249*	*6*.*5*						
**Group of age**								
18–24 years	13	10.3	1					
25–34 years	42	12.1	1.17	(0.65–2.11)	0.594	1.45	(0.78–2.69)	0.235
35–44 years	65	8.2	0.79	(0.45–1.40)	0.425	1.02	(0.56–1.87)	0.947
45–54 years	72	6.8	0.65	(0.37–1.15)	0.139	0.84	(0.46–1.51)	0.551
55–64 years	37	4.1	**0.40**	**(0.22–0.72)**	**0.003**	**0.50**	**(0.27–0.94)**	**0.032**
≥65 years	20	3.4	**0.33**	**(0.17–0.64)**	**0.001**	**0.42**	**(0.21–0.82)**	**0.012**
**Gender**								
Man	76	6.6	1					
Woman	173	6.4	0.97	(0.75–1.26)	0.817			
**educational level**								
No education	2	20.0	3.41	(0.98–11.90)	0.054	**4.11**	**(1.37–12.34)**	**0.012**
Primary education	11	7.1	1.22	(0.68–2.20)	0.509	1.75	(0.97–3.15)	0.061
Secondary education	66	8.6	**1.47**	**(1.12–1.93)**	**0.006**	**1.65**	**(1.24–2.19)**	**0.001**
University education	170	5.9	1					
**member of a risk group**								
Yes	31	3.6	1					
No	192	7.5	**2.09**	**(1.44–3.04)**	**<0.001**	**1.69**	**(1.16–2.46)**	**0.007**
No, but living with someone at-risk	26	6.5	1.83	(1.10–3.04)	0.020	1.48	(0.89–2.45)	0.128

## Discussion

On September 30, 2021, a total of 36.6 million people were fully vaccinated in Spain (77.1%), and 37.6 million had received at least one dose (79.4%) [12, 40]. To achieve pandemic control, it is necessary for vaccines to be available in easily accessible immunization services, and that the population understands the need and value of vaccination [41]. However, vaccine hesitation, defined as the delay in accepting or refusing vaccination despite availability, can become the main obstacle to these prevention efforts [42].

Behind hesitation, there is a set of factors of different natures [43, 44]. To address the hesitation over the COVID-19 vaccine, an analysis of such factors is needed to further assess the scope and magnitude of this public health threat [27, 45].

The present study sought to provide an investigation of attitudes towards vaccines and intention to vaccinate against COVID-19 according to sociodemographic characteristics after the approval of the vaccine in Spain. With a sample size of 4,352 individuals (of whom 11.53% were HP), we have a good representation of different age groups, gender, educational level and membership of risk groups. At the time of launching our study, the vaccination process according to the Spanish’s COVID-19 vaccination strategy was between Stage 2 (focus on the vaccination of people over 50 years of age) and Stage 3, which started in June 2021 and focused on vaccinating the remaining age groups (from 49 years old to 5 years old) [12]. During our survey period, the number of people who received the full plan (two doses) increased from 4.6 million to 28.5 million people, and the number of people who received at least one dose increased from 11.7 million to 33.4 million people [12]. In this scenario, we found that the acceptance rate in the total sample was 87%. If we compare this result with some studies carried out in the Spanish population just before the vaccine was available, we notice that the degree of reluctance to be vaccinated has decreased notably, from 13.4–26.8% before starting the vaccination campaign to 6.5% 6 months after the beginning of the COVID-19 mass vaccination campaign [18–20]. Despite this, these results should be viewed with caution, as the samples of the different studies may not be comparable due to the sampling method. A possible explanation for the decrease in the COVID-19 vaccine rejection could be that when the proportion of vaccinated people increases, possible misbeliefs about the vaccine are also dispelled due to the lack of evident side effects and the observable decrease in mortality among those vaccinated. Our results seem to be in line with those of the United Kingdom, where indecision has been decreasing from January 2021 to July 2021 [46]. The proportion of vaccine hesitancy in the United Kingdom was about 4% during our study period, which was slightly lower than the one we found and also decreased with age [47]. The Spanish’s COVID-19 vaccination strategy followed could explains why the prevalence of vaccination in the GP was higher with increasing age and that most of the HP were already vaccinated [12].

Hesitation about vaccination can be seen as a threat to global health, especially if it is the HP who display negative attitudes towards vaccination. A systematic review that collected data through February 2021 found that vaccine acceptance among HP varied broadly, ranging from 27.7% to 77.3% [48]. Although our data have shown low levels of COVID-19 vaccine refusal in this population in Spain (above 96% of physicians and above 94% of nurses were vaccinated), vaccine hesitancy among HP is of particular concern considering their front-line position in the fight against the spread and impact of the COVID-19 pandemic, as it exposes them to an increased risk of infection [49, 50]. Despite the low number of HP in our sample, these results were similar to other studies carried out in parallel in Spain [51].

The majority of respondents from Spain did not have a definite preference for which vaccine they would like to be vaccinated with, and the attributes of their preferences for vaccines varied–as did the factors that influenced the hesitancy. However, there was a significant percentage of respondents who preferred to be vaccinated with mRNA vaccines over ChAdOx1 as the best option, being these results consistent with an study conducted in Spain, France, Italy and Germany [52]. This could be due to the reports highlighting problems with side effects with some inactivated vaccines, information spread not only by denialist and anti-vaccine groups but also by global media (newspapers, tv news) and also by social media [53, 54]. The discrediting of ChAdOx1 in Spain as well as in several countries of the European community was due to a mistrust campaign both in the open media and in social networks [55, 56]. However, the same did not occur in the United Kingdom, probably due to nationalistic sentiment, as the vaccine was created by Oxford University, reinforced by a large political campaign and the lack of severe complications observed during the massive vaccination efforts. Nevertheless, although the blood clot scare did not reduced confidence in vaccines in general, it affected the United Kingdom GP’s preference for ChAdOx1 vaccine [57].

In general, only 6.8% GP and 11.1% HP in our study exhibited ideas that aligned to conspiracy movements, which is the population traditionally most difficult to convince with data [58]. However, the results obtained in relation to the HP should be taken with caution given the low number that answered the survey.

It is surprising that, despite the high acceptance of the vaccine, GP Spanish respondents were more concerned about the safety and efficacy of the vaccine (above 52% of GP who had reticence to be vaccinated against COVID-19). 17.3% refused to get vaccinated because they believe is better to naturally generate antibodies by surviving infection. No statistically significant differences were found between gender in relation to negative attitudes toward vaccines.

Finally, attitudes of lack of trust towards vaccination were higher among people aged 18–24 years in comparison with people older than 55 years, those with a lower level of education and people who were not part of a risk group. This is probably due to the fact that young people perceive less risk in the face of this disease. Lack of education may also influence the information available about vaccines or how it is processed. Previous studies have shown that only 12.6% of respondents stated that they would not be willing to receive a COVID-19 vaccine [22, 59, 60].

These results are useful for the design of effective vaccination promotion strategies and immunization coverage programs for the public to build confidence in COVID-19 vaccination among the public [61] such as taking steps to disseminate clear and timely information, with specific and personalized messages with key recommendations for the general population, through reliable channels to promote its safety and effectiveness, and to achieve high vaccination coverage in the vaccination schedule [62]. Understanding people’s behaviour when facing large vaccination campaigns should allow us to improve the acceptance rate, to whom these campaigns should be directed, and with what channels of diffusion should be used.

Our research has several strengths and limitations. First, although this study is not randomized, its results are based on a large convenience sample and it is one of the first studies in which both Spanish population and HP have participated. Despite the low response rate among HP, in the end, we obtained the assessment of 502 representatives of physicians, nurses and other HP. Consequently, the results must be interpreted and considered in light of this. Second, as a survey-based study, all data were self-reported by the participants, which may reduce certain response biases, such as social desirability. The questionnaire was designed adhoc for this study and the questions were developed by a group of experts based on previous research and normative questions, and despite it was not validated to determine vaccination intention and attitude toward COVID-19 vaccination, since in no case was it intended to measure any abstract construct but rather to learn about perceptions, we believe that the survey questions we posed were pragmatic in nature and the responses accurately reflected the sentiment of all groups. And finally, the questions in the questionnaire were prioritized with the aim of the study in mind so that they could be answered within a maximum of 2 minutes. Other potentially relevant variables that may influence vaccine acceptance or refusal, such as the exact place of origin, comorbidity burden, political ideology, cultural and religious values, medical specialty, etc., could be collected. Nevertheless, we thought that an increase in the time required to answer the questionnaire would have been associated with a lower response from the target population.

## Conclusions

The acceptance of the vaccination against the coronavirus was not affected by the anti-vaccine movements or by the misinformation of some media, being the youngest people and with a lower educational level those who presented a greater hesitancy, probably considering that they presented a lower risk than older adults. In addition, health professionals did not reject vaccination in a significant way, although they did have more concerns about the safety and efficiency of the vaccines. These benefits can be evidenced by showing that when pro-vaccination campaigns are given, hesitancy decreases as can be evidenced in the results, presumably due to a better understanding of the use and benefits of the vaccine against COVID-19.

## Supporting information

S1 File STROBE statement—Checklist of items that should be included in reports of cross-sectional studies. (PDF)

S2 File Survey addressed to healthcare professionals. (PDF)

S3 File Survey addressed to general population. (PDF)
