# Supplementary material for: Hesitation about coronavirus vaccines in healthcare professionals and general population in Spain
Source: PLoS One. 2022 Dec 1;17(12):e0277899. doi: 10.1371/journal.pone.0277899 (PMC9714825; doi:10.1371/journal.pone.0277899)
Supplement: S3 File — (PDF) [file pone.0277899.s003.pdf]

WELCOME TO THE COVID19 VACCINE SURVEY

We would appreciate your collaboration in filling out this short questionnaire about the COVID-19 vaccine. This is a research project developed by the Universitat Oberta de Catalunya (UOC) through the eHealth Center, with the aim of studying the opinion of the Spanish population on the COVID-19 vaccine.

The purpose of the research project and the purpose of the data processing is to contribute to the reorientation of health strategies on COVID-19 vaccination.

Personal data collected with the express consent of the person concerned will be pseudonymized and statistical methods will be applied to aggregate the primary data and obtain results for the final report and scientific publications that may be produced. Your data will not be communicated to third parties, nor is the international transfer of data foreseen. The primary data will be kept for the time necessary to fulfill the purpose for which they were collected.

Your input is essential. Please do not hesitate to forward the survey to your friends and relatives.

Thank you very much for your attention and interest.

Responding to the survey will take approximately **1 minute of your time**.

Based on the information provided, I confirm that:

I have understood the information provided to me about the objective and my participation in it.

I have understood the information regarding data protection.

In which Spanish Autonomous Community do you reside?

Andalucía

Aragón

Principado de Asturias

Illes Balears

Canarias

Cantabria

Castilla y León

Castilla-La Mancha

Cataluña

Comunitat Valenciana

Extremadura

Galicia

Comunidad de Madrid

Región de Murcia

Comunidad Foral de Navarra

País Vasco

La Rioja

Ciudad Autónoma de Ceuta

Ciudad Autónoma de Melilla

How old are you?

From 18 to 24 years old

From 25 to 34 years old

From 35 to 44 years old

From 45 to 54 years old

From 55 to 64 years old

65 and over

What is your gender?

Men

Woman

Other:

What is the maximum level of education completed?

No education or unfinished primary education

Primary school or school certificate

Secondary (BUP, COU, baccalaureate; FP2, vocational training)

University graduates

Do you belong to the at-risk group?

Yes

No

No, but I live with someone who does belong to the at-risk group.

Have you been vaccinated against COVID-19?

Yes

No

What is the vaccine you have been vaccinated with?

Astra Zeneca

Pfizer

Modern

Sputnik

Johnson & Johnson

Other:

Is it the vaccine that you wanted to be vaccinated?

Yes

No

I didn't care

If you would like to add any comments, you may do so now.

Do you plan to be vaccinated against COVID-19?

Yes

No

Which vaccine do you think is better?

Astra Zeneca

Pfizer

Modern

Sputnik

Johnson & Johnson

I don't care

Other:

If you want to add a comment, you can do it now.

Why don't you plan to get vaccinated against COVID-19?

COVID-19 is not a real disease. I don't believe in the pandemic, it is a lie created by governments/pharma to make money, there are other better alternatives.

COVID is a new disease, and the vaccine has not yet been proven to work/be effective.

I am concerned about the unknown serious effects of the vaccine in the future.

I feel that vaccination is not necessary, better to get sick and generate antibodies

Natural immunity lasts longer than vaccination

Natural exposure to disease is safer for the immune system than vaccination

Natural exposure to germs and viruses provides the safest protection.

Religious reasons

Other:

If you would like to add any comments, you may do so now.

Click the arrow below to finish and submit the survey

Powered by Qualtrics
